# Treatment of Dystonic Tremor of the Upper Limbs: A Single-Center Retrospective Study

**DOI:** 10.3390/jcm12041427

**Published:** 2023-02-10

**Authors:** Belén González-Herrero, Ilaria Antonella Di Vico, Erlick Pereira, Mark Edwards, Francesca Morgante

**Affiliations:** 1 Neurosciences Research Centre, Molecular and Clinical Sciences Institute, St. George’s University of London, London SW17 0RE, UK; 2 Departamento de Medicina, Universidad Autónoma de Barcelona (UAB), 08193 Barcelona, Spain; 3 Neurology Unit, Movement Disorders Division, Department of Neurosciences Biomedicine and Movement Sciences, University of Verona, 37134 Verona, Italy; 4 Department of Clinical and Basic Neuroscience, Institute of Psychiatry, Psychology and Neuroscience, King’s College London, London SE5 8AF, UK; 5 Dipartimento di Medicina Clinica e Sperimentale, University of Messina, 98122 Messina, Italy

**Keywords:** dystonia, tremor, dystonic tremor, botulinum toxin, deep brain stimulation

## Abstract

Tremor is part of the phenomenological spectrum of dystonia. Treatments available for tremor in dystonia are oral medications (OM), botulinum neurotoxin (BoNT), and brain surgery (deep brain stimulation or thalamotomy). There is limited knowledge regarding the outcome of different treatment options, and evidence is especially scarce for the tremor of the upper limbs occurring in people with dystonia. In this single-center retrospective study, we evaluated the outcome of different treatments in a cohort of people with upper limb dystonic tremors. Demographic, clinical, and treatment data were analyzed. Dropout rates and side effects were specifically assessed, as well as the 7-point patient-completed clinical global impression scale (p-CGI-S, 1: very much improved; 7: very much worse) as outcome measures. A total of 47 subjects (46.8% female) with dystonic tremor, tremor associated with dystonia, or task-specific tremor were included, with a median age at onset of 58 years (7–86). A total of 31 subjects were treated with OM, 31 with BoNT, and 7 with surgery. Dropout rates with OM were 74.2% due to either lack of efficacy (*n* = 10) or side effects (*n* = 13). A total of 7 patients treated with BoNT (22.6%) had mild weakness, causing dropout in 2. P-CGI-S was ≤3 (improvement) in 39% with OM, compared to 92% with BoNT and 100% with surgery. These findings suggest good symptom control of the tremor of the upper limb in dystonia with BoNT and surgery, with higher rates of dropout and side effects with OM. Randomized controlled studies are needed to confirm our findings and provide further insight into better selecting suitable patients for BoNT or brain surgery.

## 1. Introduction

Tremor is an involuntary, rhythmic, oscillatory movement of a body part [1], and it is one of the phenomenological manifestations of dystonia. Tremor in dystonia is classified into two types: dystonic tremor (Dys-T), which occurs in body parts affected by dystonia, and tremor associated with dystonia (TAD) that appears in a body part not affected by dystonia in a person with dystonia located elsewhere. Classically, tremor in dystonia (whether Dys-T or TAD) is variable in frequency and amplitude and exacerbated in specific positions. While generally a postural and kinetic tremor, it may also occur at rest and is frequently asymmetric [2,3]. Task-specific tremor (TST) is a form of action tremor that occurs only or mostly when performing a specific skilled task [4], most commonly seen during writing, called primary writing tremor (PWT). Although the pathophysiology of TST is still debated, growing data favors its dystonic nature [5,6].

There are no formal guidelines for the treatment of Dys-T, TAD, and TST. In a recent placebo-controlled, parallel-group, randomized clinical trial [7] conducted on 30 subjects with dystonic hand tremor, onabotulinum toxin A significantly improved the Fahn–Tolosa–Marin Tremor Rating Scale total score. A systematic review of the available data on the treatment of Dys-T and PWT concluded that botulinum neurotoxin (BoNT) and functional neurosurgery (deep brain stimulation, DBS, and thalamotomy by radiofrequency) might be more effective than oral medications; however, there was no data on the treatment of upper limb Dys-T with BoNT and no data at all for TAD [8]. Moreover, there are no long-term follow-up data regarding the treatment of different types of tremors in dystonia. 

The present retrospective study aimed to evaluate the short and long-term clinical outcomes of people with Dys-T, TAD, and TST affecting the upper limbs and treated with oral medications, BoNT, and functional neurosurgery in a tertiary referral center for movement disorders.

## 2. Methods

This is a retrospective cohort study including consecutive patients diagnosed with Dys-T, TAD, or TST affecting the upper limbs at the Movement Disorders Clinic at St. George’s Hospital, London, UK, between August 2016 and October 2021. 

Medical records of all participants were systematically reviewed and included all documents available from two movement disorder specialists (FM and ME) at St. George’s Hospital, London. Patients were excluded from the study if comprehensive medical records throughout follow-up were unavailable. We included all patients whose upper extremity tremor was classified as an idiopathic dystonic tremor and excluded individuals whose tremor was considered secondary. 

Demographic and medical history data were recorded, including sex, age at the onset of tremor, duration at first assessment, follow-up duration, and family history of movement disorders. 

Clinical examination was standardized and included the following aspects: assessment at resting, postural and kinetic tremor with eyes open and closed; and assessment of tremor while performing different manual tasks, including writing, using cutlery, holding a cup, typing on a keyboard and cell phone, or playing an instrument. The following clinical features were retrieved: type of tremor (Dys-T, TAD and TST), distribution (restricted to the upper limbs or present in other body districts), side of tremor, if asymmetric, presence of resting component, and predominant pattern of tremor. Patients were also asked about the daily tasks most impacted by the tremor. 

Data on oral treatment included the number and type of medications prescribed, side effects, and dropout rates due to inefficacy. Inefficacy was determined when patients had been on the maximum dose for at least six months, without benefit. All patients undergoing surgical and botulinum toxin treatment were treated by the same movement disorder specialist (FM). Electromyography and/or electrical stimulation guidance was used when injecting botulinum toxin whenever clinical examination did not disclose a consistent pattern of tremor, or to inject fingers, forearm flexors, or extensors. 

Incobotulinum toxin A (Merz Pharma GmbH & Co. KGaA, Frankfurt, Germany) was prepared by adding 1 or 2 mL of preservative-free saline into 100 U vials, and abobotulinum toxin A (Ipsen Biopharm limited, Wrexham, UK) by adding 2.5 mL of preservative-free saline into 500 U vials. We collected the following data on BoNT treatment: tremor duration at first injection, the number of injections received, treatment duration, injection interval, type of BoNT, the dose of BoNT at first and last injection, muscles injected, side effects, and dropout rates. 

Data from patients treated with surgery included: the type of procedure (deep brain stimulation, DBS, or radiofrequency thalamotomy), type of device, age at surgery, DBS settings, and side effects. 

A patient-completed Clinical Global Impression (CGI) rating scale was assessed for all patients, regardless of the treatment received. Our study included the modified patient-rated CGI-improvement (p-CGI-I) at the follow-up visits as an outcome measure. The scale measures improvement on a 7-point scale (1: Very much improved, 2: Much improved, 3: Minimally improved, 4: No change, 5: Minimally worse, 6: Much worse, 7: Very much worse) based on the perception of the patient regarding how much he or she had improved or worsened relative to a baseline state at the beginning of the intervention.

## 3. Statistical Analysis

Comparisons between groups were performed using Fisher’s exact test for categorical variables and the U Mann–Whitney test for continuous variables. Grading scores were analyzed using the U Mann–Whitney test and the Wilcoxon signed-rank test for ordinal variables. The results were considered statistically significant at a 2-tailed *p* < 0.05. SPSS software version 28 (IBM Corp., Armonk, NY, USA) was used to perform the analysis. All data are reported as mean ± standard deviation, unless otherwise stated. 

## 4. Results

A total of 47 patients (22 female, 46.8%) were included in the study, with a median (interquartile range, IQR) age at the first assessment of 70.9 (62.3–75.9) years and tremor duration of 8.8 (3.8–28.6) years. They were followed up for a median (IQR) of 24.8 (5.3–35.6) months. 

A total of 33 patients had Dys-T (70.2%), 4 patients had TAD (8.5%), and 10 TST (21.3%). The tremor was postural or kinetic in all the patients, with 4 (8.5%) also having a rest component. The tremor was typically restricted to the upper limbs (70.2%), bilateral (66%), and asymmetric (96.8%). Complete demographic and clinical data are presented in Table 1. 

A total of 43 patients (91.5%) received treatment of multiple types; 4 did not, due to personal preference or mild presentation. Throughout the period of follow-up, 31 (72.1%) received oral medications, 31 (72.1%) BoNT, and 7 (16.3%) underwent surgery (6 DBS, 1 thalamotomy).

### 4.1. Treatment with Oral Medication

**Figure 1:**
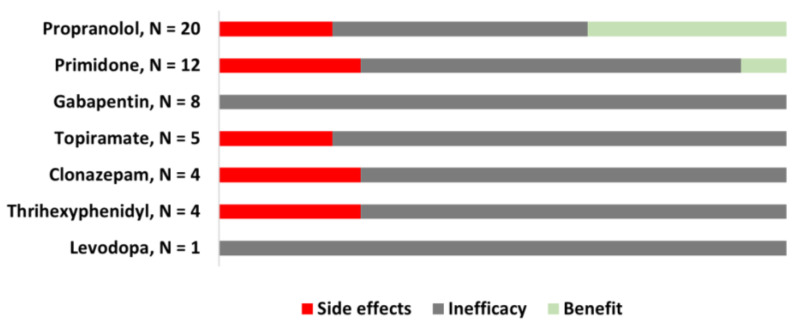
*Effect of oral medications for dystonic tremor of the upper limbs.* The most frequently prescribed oral medications for tremor were propranolol (64.5%), primidone (38.7%), and gabapentin (25.8%). Benefits were only reported with propranolol (35%) and primidone (8%). Topiramate, clonazepam, trihexyphenidyl, and levodopa, were either ineffective or caused side effects (topiramate in 20%, clonazepam in 25%, and trihexyphenidyl in 25%).

A total of 31 patients (72.1%) were treated with at least one oral medication (1.74 ± 1.1). Considering patients utilizing all oral medications together, 23 (74.2%) dropped out due to either side effects (41.9%), most commonly drowsiness and lightheadedness, or inefficacy (32.3%). Still, despite some improvement, 5 patients required combined therapy with BoNT (4, 12.9%) or surgery (1, 3.2%) to achieve satisfactory benefits. Individualized benefits and side-effect percentages resulting from the different oral medications are summarized in Figure 1. 

### 4.2. Treatment with BoNT

**Figure 2:**
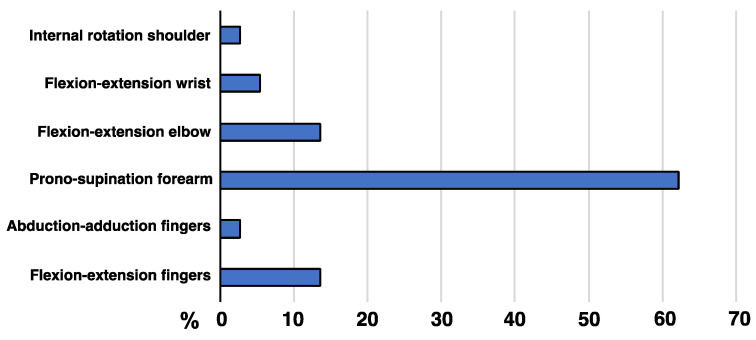
*Main pattern of dystonic tremor of the upper limbs*. Pronosupination was the most frequent tremor pattern observed while performing the most affected manual task.

The decision to treat with BoNT was made in 31 patients (72.1%), and 19 of them had taken oral medication previously, without satisfactory outcomes. Out of 31 subjects, 26 (83.9%) were still receiving this treatment at the last follow-up. A total of 2 patients showed handgrip weakness and did not want to continue the treatment, 1 had an intercurrent stroke affecting the previously injected arm, and 2 patients were lost to the follow-up. Figure 2 reports the main tremor pattern during the execution of the manual tasks identified as goals of the BoNT treatment in each patient. These therapeutical goals included high dexterity activities (19.2%), holding a cup (65.4%), writing (34.5%), and using cutlery (19.2%).

The median (IQR) tremor duration at first injection was 8.8 (4.9–23.7) years, and the median (IQR) treatment duration with BoNT was 27.7 (4.8–34.5) months. The mean interval within injections was 4.8 ± 1.7 months. EMG and/or electrical stimulation were employed to optimize the injection in 8 subjects (25.8%).

Abobotulinum and incobotulinum toxins were similarly used (57.5% vs. 42.3%, respectively). Patients underwent, on average, 4.5 ± 3.3 injection sessions. Table 2 shows the muscles injected. The incobotulinum toxin dose was significantly higher at the last injection (68.6 ± 35.7) compared to the first injection (50.8 ± 26.1) (*p* = 0.03). The dose of the first (271.3 ± 196.8) and last injection (250 ± 164.1) with abobotulinum toxin was comparable (*p* = 0.43).

A total of 7 subjects (22.6%) exhibited transitory weakness (4 with abobotulinum toxin, causing dropout in one, and 3 with incobotulinum toxin, causing dropout in one).

The duration of side effects was comparable between the two formulations of BoNT (abobotulinum toxin: 20 (IQR, 10–90) days; incobotulinum toxin: 20 (IQR, 7–60) days, U Mann–Whitney *p* = 0.4).

### 4.3. Treatment with Functional Neurosurgery

A total of 7 patients (14.9%) were treated with brain surgery, 6 with DBS, and 1 with unilateral radiofrequency thalamotomy in the VIM nucleus. St. Jude Medical Infinity directional DBS leads (Abbott Neuromodulation, Austin, TX, USA), spaced at 1.5 mm apart, were implanted bilaterally in all patients receiving DBS. The ventralis intermedius (VIM) nucleus of the thalamus and caudal zona incerta (cZI) were dual-targeted using classic anterior commissure, posterior commissure (ACPC) stereotactic coordinates (x = +/−13, y = −4, z = 0). The tips of the electrodes were positioned in the cZI (posterior subthalamic area). The rationale for deciding to use unilateral thalamotomy instead of DBS in the 1 patient was age (she was over 80 years old), and the tremor predominantly affected the right side of the body. She had some balance difficulties predating the surgery, and her expectations were gaining independence in the basic activities of daily living, such as holding cutlery and holding a stick when walking. The increased risk of bilateral DBS surgery and the unnecessary burden of attending subsequent programming sessions favored thalamotomy over DBS.

All patients treated with brain surgery had previously received treatment with oral medications, without benefit. None of them had received BoNT injections before surgery. The average age at surgery was 70.8 ± 8.5 years, and the median (IQR) tremor duration was 23 (9–42) years. The follow-up median (IQR) time was 29.1 (14.9–47.9) months.

A total of 2 patients had chronic stimulation-induced side effects (dysarthria and balance disturbances). The patient with thalamotomy exhibited balance disturbances predating surgery, which transitorily worsened after surgery. She recovered and reached her baseline three weeks after surgery. Clinical data and DBS settings at the last follow-up are collated in Table 3.

### 4.4. Clinical Global Improvement (CGI-I)

**Figure 3:**
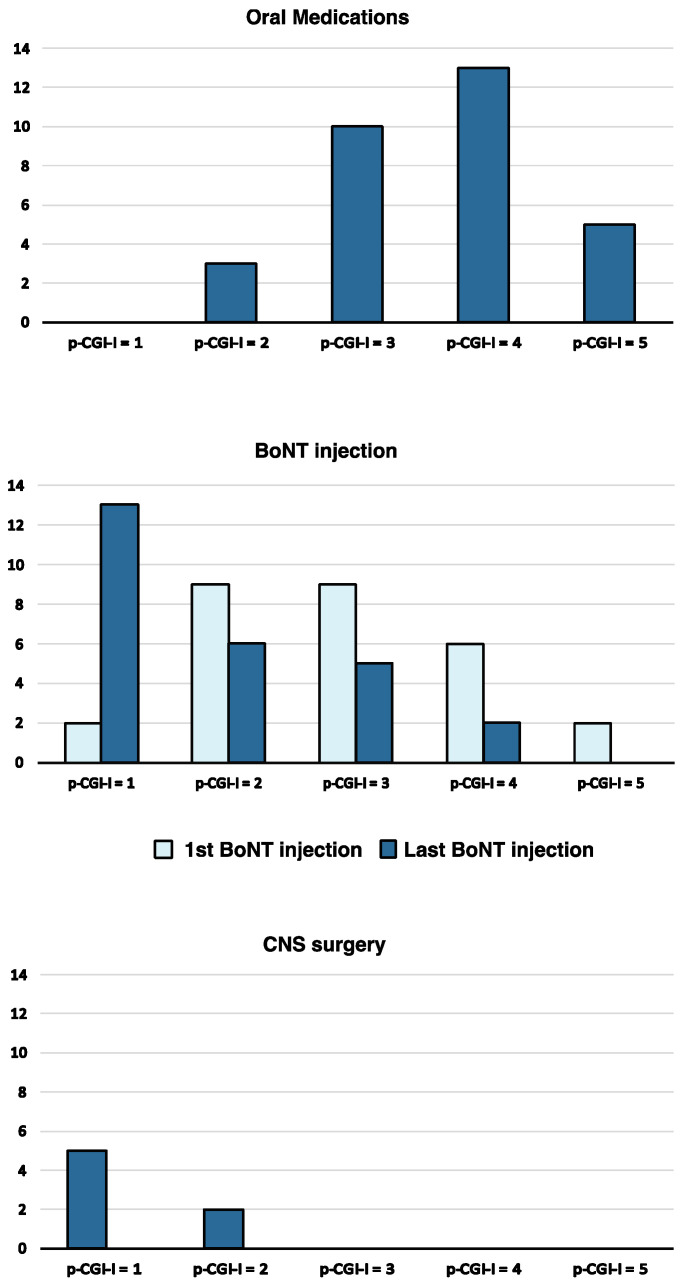
*Patient-based Clinical Global Improvement (p-CGI-I) as per treatment for tremor*. More patients reported improvement on the p-CGI-I with botulinum toxin (BoNT) injections and neurosurgery compared to oral medications. The percentage of p-CGI-I ≤ 3 was significantly higher when comparing the first and last injection of BoNT (74% vs. 92%, Fisher’s exact test *p* = 0.04). P-CGI = 1: Very much improved, 2: Much improved, 3: Minimally improved, 4: No change, 5: Minimally worse, 6: Much worse, 7: Very much worse.

The patient-reported outcomes with the different therapies are represented in Figure 3. With oral medication, 40% of patients reported a p-CGI-I ≤ 3 (improvement) compared to 92% after optimized treatment with BoNT (at the last injection) and 100% after surgery. The percentage of p-CGI-I ≤ 3 was significantly higher when comparing the first and last injection of BoNT (71% vs. 92%, Fisher’s exact test; *p* = 0.04). There were no significant differences in p-CGI scores in regards to botulinum toxin type (Mann–Whitney U test; *p* = 0.82) or the usage of EMG guidance (*p* = 0.4).

In the group of patients that received both oral medications and BoNT at any time during the entire follow-up (16, 37.2%), the pCGI-I was significantly higher with BoNT compared to oral medications (Wilcoxon Rank Squares; *p* = 0.001).

## 5. Discussion

There is an unmet need for evidence-based guidelines to treat tremor in dystonia [8]. The different criteria and labeling used by clinicians for isolated upper limb tremors and the lack of biomarkers to distinguish between tremor syndromes have made it difficult to draw definitive conclusions about how best to treat dystonic tremors of the upper limb.

Here, we reviewed three very different approaches to treating patients with tremor in dystonia: oral medication, BoNT, or brain surgery. While oral medication has a systemic effect, BoNT is administered by local intramuscular injections in the muscles, and deep brain stimulation or thalamotomy require brain surgery. Oral medications for tremor more commonly produce sickness, lightheadedness, or drowsiness and may be contraindicated if the patient has other comorbidities or if there is interaction with other medications; BoNT can produce bruising and weakness in the injected muscles, and brain surgery can result in a hemorrhagic stroke or a brain infection, to side effects ultimately related to the stimulation, more commonly in the target for tremor, balance, and speech disturbances.

Our study suggests that commonly used oral medications for tremor are less effective than BoNT and surgery in treating any type of tremor of the upper limbs occurring in people with dystonia and task-specific tremors. Dropout rates and side effects were higher with oral medications. BoNT appeared to be an effective and safe therapy, leading to improvement in 71% of the patients after the first injection; this percentage increased to 92% when the injection protocol was optimized at subsequent follow-up visits. In patients who received both oral medications and BoNT at any time during their follow-up, improvement based on the p-CGI-I was significantly greater with BoNT. These results are in keeping with those of Fasano et al. in their systematic review [8], in which they found BoNT to be superior to oral medication for axial dystonic tremor and task-specific tremor, as well as with the results from the placebo-controlled trial from Rajan et al. [7], in which BoNT significantly improved the outcome of 30 subjects with dystonic hand tremor.

The outcome of any tremor treatment is often assessed with validated rating scales developed for essential tremor, such as The Fahn–Tolosa–Marin [9] or the Tetras scale [10], as well as instruments measuring the quality of life, such as QUEST [11]. However, such outcome measures were not designed for subjects whose tremor occurs in the context of dystonia or for those having a purely task-specific tremor. In our study, from medical history and clinical examination, we identified the manual tasks most affected by tremor, similar to the goal attainment scale employed in spasticity studies [12]. The p-CGI was based on their satisfaction with the treatment regarding the manual task most impacted by the tremor, which was most commonly holding a cup. This also allowed us to identify the pattern of tremor (clinically or by EMG) while executing that specific action and injecting the botulinum toxin accordingly. Most frequently, there was a pronosupination of the forearm and flexion-extension at the elbow. Therefore, the most injected muscles were the supinator longus, pronator teres, biceps, and triceps (Table 2).

Previous studies using BoNT in the treatment of upper limb tremor only injected either the wrist flexors/extensors [13,14] or selected the muscles by the clinical and electrophysiological assessment of patients with arms at rest, outstretched in front of the subject, and while performing finger-to-nose action [7,15]. Injecting the wrist extensors increases the possibility of handgrip weakness [14,16], and typically, tremor in dystonia changes with different positions. This makes it imperative, in our opinion, that the selection of muscles for injection takes place in reference to specific task performance. Given our retrospective data, prospective controlled studies are warranted to test this approach in subjects with Dys-T, DAT, or TST.

Deep brain surgery was the most effective treatment for those failing first, second, and third-line oral medications for tremor. None of the patients treated with brain surgery had received BoNT. They were selected for brain surgery based on the severity of the tremor, which determined functional impairment and significant disability in the absence of exclusion criteria. Data on DBS for tremor in dystonia are scanty, with different targets proposed in addition to the globus pallidus pars interna^11^, such as the VIM, ventralis oralis anterior^12^, and caudal ZI^13^. Our cohort was implanted, positioning the most inferior contact in cZI/PSA, allowing for the targeting of two different structures (cZI and VIM), previously demonstrated to be effective for Dys-T [17].

Several limitations of this study should be considered. The results should be cautiously interpreted, as the data were analyzed retrospectively, and the sample number is relatively small in some analyses. Treatment decisions were based on clinical expertise and patients’ preferences. Moreover, some data were not included due to incomplete reports. A standardized dystonia rating scale was not employed to formally assess tremor and dystonia symptoms. A clinical examination was not always accompanied by EMG or kinematic assessment. Lastly, the p-CGI-I is a subjective measure of improvement based on the patient’s perception, which could have been supplemented by objective measures.

Despite these limitations, our data suggest that oral medications employed for essential tremor are often ineffective or not tolerated in people with dystonic tremors of the upper limbs. Botulinum toxin appears to be a safe and effective therapeutic option that warrants testing in randomized controlled trials with open-label, long-term follow-up. This seems to be particularly true if the tremor has a predominant pattern of pronosupination of the forearm or flexion-extension at the elbow when performing the most-affected manual tasks. Therefore, there is a need to develop specific rating scales for dystonic tremors that also consider essential manual tasks commonly affected. Finally, functional neurosurgery also seems to be a very effective procedure. Large randomized controlled trials are needed to confirm our findings and provide further insight into selecting patients best suited to receive BoNT, DBS, or thalamotomy.

## Figures and Tables

**Table 1:** Demographic and clinical data of subjects with dystonic tremor, tremor associated with dystonia, and task-specific tremor.

	*n* = 47
**Sex (*n*, %)**	
Male	25 (53.4)
Female	22 (46.8)
**Family history of movement disorders (*n*, %)**	
Negative	33 (70.2)
Tremor	13 (27.7)
Parkinsonism	1 (2.1)
**Age at onset, median (IQR), years**	58.0 [41.5–67.7]
**Age at first assessment, median (IQR), years**	70.9 [62.3–75.9]
**Total time follow-up, median (IQR), months**	24.8 [5.3–35.6]
**Tremor duration at first assessment, median (IQR), years**	8.8 [3.8–28.6]
**Type of tremor (*n*, %)**	
Dystonic tremor (Dys-T)	33 (70.2)
Tremor associated with dystonia (TAD)	4 (8.5)
Task-specific tremor (TST)	10 (21.3)
**Body districts involved (*n*, %)**	
Only UL	13 (27.7)
UL + Legs	3 (6.4)
UL + Head/Neck	11 (23.4)
UL + Oromandibular	1 (2.1)
UL + Voice	4 (8.5)
**Laterality (*n*, %)**	
Monolateral	31 (66)
Bilateral	16 (34)
Asymmetric	30 (97.9)

Data are presented as numbers (*n*, %) for categorical variables and median (interquartile range, IQR) for continuous variables. Dys-T = dystonic tremor; TAD = tremor associated with dystonia; TST = task specific tremor; UL = upper limbs.

**Table 2:** Type of botulinum toxin, injected muscles, and botulinum toxin dose.

		Abobotulinum Toxin/A (Units)	Incobotulinum Toxin/A (Units)
**Muscles injected (*n*, %)**	*n* = 31		
Pronator teres	18 (58.0)	78.0 ± 26.9	23.1 ± 9.6
Longus supinator	21 (67.7)	76.7 ± 26.7	22.8 ± 9.0
Biceps	8 (25.8)	83.7 ± 41.4	30.0 ± 10.0
Triceps	11 (35.5)	101.7 ± 28.6	20 ± 0.0
Flexor carpi radialis	3 (9.7)	20	30 ± 14.14
Flexor carpi ulnaris	3 (9.7)	55 ± 7.0	30
Extensor carpi radialis	2 (6.4)	-	30 ± 20
Teres major/minor	3 (9.7)	80 ± 40	-
Flexor superficialis digitorum	3 (9.7)	40	30 ± 0.0
Flexor profundus digitorum	3 (9.7)	-	30 ± 17.32

Data are presented as numbers (*n*, %) for categorical variables and mean ± SD for continuous variables. The most frequently injected muscles were the pronator teres, longus supinator biceps, and triceps.

**Table 3:** Subjects with dystonic tremor and task-specific tremor treated with functional neurosurgery.

Gender/Age	Type of Tremor	Age at Onset (Years)	Disease Duration at Surgery (Years)	DBS Settings	Side Effects with Current DBS Settings or after Thalamotomy
Male, 63	Dystonic Tremor	20	43	Left: 1-, case+, 60 mcs,130 Hz, 2 mA Right: 9-, case+, 60 mcs,130 Hz, 2.7 mA	Mild stimulation induced dysarthria and balance difficulties.
Male, 66	Dystonic Tremor	57	9	Left: 2(abc)-, case+, 60 mcs, 130 Hz, 2.6 mA Right: 9-, case+, 60 mcs, 130 Hz, 1.4 mA	None
Male, 73	Dystonic Tremor	50	23	Left 1-, 2(abc)+, 50 mcs, 170 Hz, 2.5 mA Right 9-, 10(abc)+, 60 mcs, 170 Hz, 3.0 mA	None
Male, 70	Dystonic Tremor	60	10	Left 2a-, case+, 60 mcs, 130 Hz, 4.0 mA Right 11c-, case+, 60 mcs, 130 Hz, 3.0 mA	None
Female, 60	Task-specific Tremor	56	4	Left 3c-, case+, 60 mcs, 190 Hz, 3.5 mA, Right 12-, case+, 60 mcs, 130 Hz, 2.1 mA	None
Female, 81	Dystonic Tremor	7	74	Left 1-, case+, 60 mcs, 130 Hz 3.4 mA Right 9-, case+, 30 mcs, 190 Hz, 4.0 mA	Mild stimulation induced dysarthria and balance difficulties.
Female, 82	Dystonic Tremor	50	32	Not applicable	Mild balance disturbance (present before surgery).

DBS = deep brain stimulation; mcs = microseconds; Hz = hertz; mA: milliampere. Subjects 1–6 were treated with DBS. Subject 7 was treated with a thalamotomy.

## Data Availability

Supporting data is not publicly available.
